# DEVELOPMENT OF A DECISION SUPPORT FRAMEWORK TO PROMOTE COMFORT AMONG PATIENTS WITH SEVERE DEMENTIA IN THE HOSPITAL

**DOI:** 10.1093/geroni/igad104.1780

**Published:** 2023-12-21

**Authors:** Nathan Davies, Ellen McCoy Smith, Malvi Shah, Elizabeth Sampson

**Affiliations:** University College London, London, England, United Kingdom; South West London and St George’s Mental Health NHS Trust, London, England, United Kingdom; University College London, London, England, United Kingdom; University College London, London, England, United Kingdom

## Abstract

Hospital wards can be challenging settings, particularly for those with dementia, and exacerbate symptoms and behavioural issues. Improving care for persons with dementia and providing a simple decision support framework to help guide staff in person-centred and holistic management of these problems has the potential to improve hospital care. We aimed to co-design an evidence-based, practical way to support hospital staff and family carers in identifying discomfort and distress, maximising comfort and reducing behaviours that challenge or are distressing to people with dementia who are towards the end-of-life. We conducted a co-design study informed by data from 1. Literature review; 2. Cohort study of comfort and discomfort in people with severe dementia admitted to hospitals (n=63); and 3. Interviews with family carers and health care professionals (n=18). These data sources were synthesised and presented to key stakeholders through co-design meetings and workshops. The resulting decision support framework uses a three-stage process of: 1) assessment of comfort/discomfort; 2) consider causes of discomfort; and 3) address needs of the individual to manage the discomfort. The framework is presented in the form of a series of flowcharts that can be used easily on busy wards. We will present examples of the framework along with key findings from the cohort study. This is the first study to develop decision support for hospital staff and families addressing comfort towards the end of life. This toolkit has practical implications for everyday working on hospital wards but also a resource for planning for end-of-life care.

